# The Immobilization Effect of Natural Mineral Materials on Cr(VI) Remediation in Water and Soil

**DOI:** 10.3390/ijerph17082832

**Published:** 2020-04-20

**Authors:** Dading Zhang, Yanqiu Xu, Xiaofei Li, Lina Wang, Xuwen He, Yan Ma, Dexun Zou

**Affiliations:** 1 School of Chemical and Environmental Engineering, China University of Mining and Technology (Beijing), Beijing 100083, China; tmaczdd02@163.com (D.Z.); xuyq15871041327@163.com (Y.X.); m18850341814@163.com (L.W.); 108137@cumtb.edu.cn (X.H.); 2 College of Chemical Engineering, Beijing University of Chemical Technology, Beijing 100029, China; lixiaofei203@126.com

**Keywords:** mineral materials, Cr(VI) pollution, adsorption model, toxicity characteristic leaching procedure, soil

## Abstract

The effects of sepiolite, montmorillonite, and attapulgite on the removal and immobilization of Cr(VI) in water and soil were studied. X-ray diffraction (XRD) characterizations showed that the purities of these three mineral materials decreased in the following order: montmorillonite > attapulgite > sepiolite, and that their surface molecular bond types were similar. The adsorption potential of Cr(VI) in aqueous solutions of the three mineral materials was in the following order: sepiolite > attapulgite > montmorillonite. The adsorption mechanism for attapulgite was consistent with the Freundlich isotherm adsorption model, whereas that for montmorillonite was more consistent with the Langmuir model. Sepiolite had a good fitting effect for both isothermal adsorption models. For montmorillonite and attapulgite, a lower pH corresponded to a higher removal of Cr(VI). For sepiolite, however, the removal efficiency of Cr(VI) from an aqueous solution was the lowest at a pH of approximately 5.0. The results of the soil toxicity characteristic leaching procedure showed that, following the addition of 15% sepiolite, attapulgite, or montmorillonite to the contaminated soil, Cr(VI) concentrations in the leachates decreased by 16.8%, 18.9%, and 15.9%, respectively, and the total Cr concentrations in the leachates were reduced by 21.2%, 29.2%, and 17.6%. Of the three mineral materials, attapulgite demonstrated the highest Cr(VI) immobilization efficiency in soil. This study emphasizes the effect of attapulgite on the immobilization of Cr(VI) in soil and aqueous solutions, thus providing a theoretical basis for the potential application of natural mineral material remediation of Cr(VI)-contaminated aqueous solutions and soils.

## 1. Introduction

Chromium is mainly used in the metallurgy, electroplating, printing, textile dyeing, and papermaking industries. Poor management and disposal of Cr-containing waste residue and wastewater from production activities in these industries can lead to Cr being discharged to the environment, resulting in serious harm to the water and soil environment and further affecting the ecosysystem [1,2,3]. A Chinese national census of pollution sources and monitoring results showed 24 categories in the national economy that produce Cr-containing waste [4]. Furthermore, 1.1% of the soil in China has Cr content that exceeds national standards, as established by the National General Survey of Soil Contamination (China, 2014) [5]. Thus, the problem of Cr pollution in China’s water and soil environments is becoming increasingly significant. Chromium has carcinogenic, teratogenic, and mutagenic effects on humans and animals, and has been identified as one of the top 20 priority controlled hazardous substances by the United States Super Fund [6,7].

In natural environments, Cr exists in numerous valence states (Cr(0)–Cr(VI)) and is mainly stable as Cr(0), Cr(III), and Cr(VI) [8]. The common oxidation states in the environment, that is, Cr(III) and Cr(VI), have different properties. Fendorfer reported that, at certain pH and electron activity values, an equilibrium exists between the thermodynamically stable Cr(III) and Cr(VI) compounds [9]. Mutual conversion of same-state chemicals occurs with changes in external conditions. As an oxidant and generator of free radicals during the reduction of Cr(VI) to Cr(III) in cells, Cr(VI) has certain toxic effects on biological systems. For instance, studies have found that occupational exposure to Cr(VI) compounds can cause a variety of clinical problems, such as respiratory and skin diseases [10,11,12]. In addition, the biological toxicity of Cr(VI) in the environment is more than 100 times that of Cr(III), as well as being more capable of migration than Cr(III), which is susceptible to an external acid–base environment and precipitates as the less mobile Cr(OH)_3_ at pH > 5.5 [13,14,15]. Cr(III) participates during cellular metabolic activities and is a necessary trace element in organisms, whereas no evidence has yet indicated that Cr(VI) participates in biological metabolic activities [8]. Therefore, identifying and studying the degraded or immobilized Cr(VI) materials in the environment is crucial to mitigate Cr(VI) pollution in water and soil.

Recently, numerous studies have examined the remediation of Cr(VI)-contaminated water and soil. The main technical methods for remediation include chemical, biological, and physical methods [16]. Chemical methods are usually based on chemical reduction–oxidation and complex precipitation, which are rapid reactions. However, such methods are costly and readily cause secondary pollution to the environment [17,18]. Singh et al. (2012) remediated Cr(VI) contaminated soil using nanoscale zero-valent iron with strong reduction ability [19]. Bioremediation methods can be divided into two types: phytoremediation (e.g., Vigna mungo) and microbial remediation (e.g., indigenous microorganisms) [20,21]. Both have low economic costs and are simple to operate. Nonetheless, long-term remediation and organism adaptation during remediation remain problematic and require further research [22]. Physical methods for the remediation of Cr(VI)-contaminated water and soil mainly immobilize metal ions by adsorption onto the surface or between the layers of an adsorbent (such as illite@carbon nanocomposite or hazelnut shell activated carbon) [23,24]. The physical adsorption method is widely used because of its simple operation, stable effects, low secondary pollution, and the possibility of regeneration and reuse [25].

Among the many adsorbent materials, natural mineral materials that act as natural pollutant scavengers are abundant, inexpensive, and environmentally friendly. They have been widely studied and applied in the field of environmental remediation [26]. Natural mineral materials have large specific surface areas and unique porous channel structures, as well as numerous active groups and negative charges that can absorb heavy metal ions, thereby reducing the migration, transformation ability, and toxicity of heavy metals in soil environments [27]. Acid (HCl, HNO_3_, H_2_SO_4_, etc.) can remove impurities in minerals and increase pore spaces; in addition, it can remove cations to increase the adsorption sites of heavy metals [28]. Acid modification is the most effective modification method to enhance the adsorption capacity of heavy metals and other pollutants [29]. Montmorillonite, attapulgite, and sepiolite, as typical 2:1 phyllosilicate minerals, have already been studied and applied as common mineral materials in heavy metal removal and remediation, due to their strong adsorption capacity [30,31,32]. Padilla-Ortega et al. studied the adsorption capacity of sepiolite for the removal of heavy metal ions in aqueous solutions and reported its adsorption capacity for metal ions as follows: Cr(III) > Cd(II) > Cu(II) > Zn(II) > Ni(II) > Ag(I) [33]. Yu et al. (2017) used two types of organically modified bentonite to remediate Cr-contaminated soil and reported that modified bentonite can immobilize Cr in soil through electrostatic attraction and characteristic adsorption [34]. In addition, Yang et al. (2017) increased the specific surface area and porosity of montmorillonite, thereby enhancing its characteristic and non-characteristic adsorption capacities for Cr(VI) in soil [35]. Zhang et al. (2019) developed humic acid-modified attapulgite and used it to remove Cr(III) from water samples [36]. Further research on the effects of different mineral materials on the treatment of Cr(VI)-contaminated water and soil is necessary, as mineral materials have a variety of mineral compositions and surface properties.

Several characterization methods are used in the analysis of minerals. X-ray diffraction (XRD) can be used to analyze and judge the composition of solid crystals, as well as to identify the phase of the sample using XRD patterns. The specific surface areas and internal microporous properties of the materials are analyzed using the Brunauer–Emmett–Teller (BET) method. Fourier-transform infrared (FTIR) spectra can reflect small molecules or molecular bond stretching vibration on the surface and inside the material. These characterization methods are useful to elucidate the properties, composition and structure of materials, and in turn help analyze the differences in the ability of different minerals to adsorb and remove Cr(VI).

In this study, three natural mineral materials (sepiolite, montmorillonite, and attapulgite) were selected to explore the following objectives: (1) the composition and surface properties of the three natural mineral materials; (2) the adsorption effects of the three natural mineral materials on Cr(VI) in aqueous solutions; and (3) the effects of the three natural mineral materials on the immobilization of Cr(VI) in soil at varying pH values. This study provides guidelines for the use of natural mineral materials in the treatment of Cr(VI)-contaminated environments by achieving the above objectives.

## 2. Materials and Methods 

### 2.1. Experimental Materials

The natural mineral materials used in this experiment were montmorillonite, attapulgite, and sepiolite, which are not pure minerals. Montmorillonite (content > 93%, SiO_2_ 57%, Al_2_O_3_ 14.3%, MgO 4.5%, Fe_2_O_3_ 2.34%) was purchased from the Zhangjiakou Hengtai Company, whereas attapulgite (content > 74%, SiO_2_ 58%, Al_2_O_3_ 9.7%, Fe_2_O_3_ 5%, CaO 1.12%) and sepiolite (content > 85%, SiO_2_ 62%, MgO 22%) were purchased as ultra-fine powders from the Hengju Mineral Processing Factory in Lingshou County. The soil sample was clay, and its moisture content, moisture density, dry density, porosity, saturation and specific gravity of the soil particles were 24.8%, 1.97 g/cm^3^, 1.58 g/cm^3^, 0.42, 92.92%, and 2.74, respectively. The chemicals used in the experiment, including potassium chromate (K_2_CrO_4_), sodium nitrate (NaNO_3_), acetic acid (C_2_H_4_O_2_), hydrochloric acid (HCl), and sodium hydroxide (NaOH), were of analytical grade. Deionized water (panning water machine, 7.0 μS/cm) was used to prepare the Cr solutions and diluted samples. The soil used in the experiments was collected from the vicinity of an electroplating workshop at a demolition factory in Hefei, Anhui Province, and its Cr(VI) content was 24.5 ± 5 mg/kg. Potassium chromate was added to the retrieved soil, which was then naturally ventilated for 60 days. Table 1 lists the physical and chemical properties of the soil.

### 2.2. Experimental Methods

#### 2.2.1. Aqueous Solution Experiment

For the aqueous solution experiment, 2 L of a 0.01 M sodium nitrate aqueous solution (0.85 g sodium nitrate in 1 L of deionized water) was prepared as a background solution in a volumetric flask. Potassium chromate was prepared as a stock solution with a Cr(VI) ion concentration of 500 mg/L. Appropriate volumes of the stock solution were used to prepare experimental solutions with Cr(VI) ion concentrations of 5, 10, 20, 40, and 50 mg/L. Natural mineral material powders (500 mg) were placed in Erlenmeyer flasks, and the experimental solutions were added at a solid–liquid ratio of 1:100. The pH (ranging from 2–9) of the solution was adjusted with a small amount of dilute HCl and NaOH solution and was measured using a pH meter. The Erlenmeyer flasks were placed in a shaker at 120 rpm/min at 25 °C for 6–8 h. After the solution had stood for 30 min, the supernatant was removed using a needle tube for analysis. The Cr(VI) concentration was measured via diphenyl carbazide spectrophotometry. The total Cr concentration was detected using inductively coupled plasma–optical emission spectroscopy (ICP-OES) (5399 DV, PerkinElmer Optima, Waltham, MA, USA). All experiments were conducted in duplicate.

#### 2.2.2. Adsorption Methods

In this study, the Langmuir and Freundlich models were used to examine the surface properties and Cr(VI) adsorption capabilities of the three mineral materials in solution. The Langmuir isotherm adsorption model has been successfully demonstrated to fit the adsorption behaviors of heavy metals in adsorbents [37,38]. The model is equivalent to a linear Equation (1) with 1/*Q_e_* as the abscissa and 1/*C_e_* as the ordinate:

(1) $$\frac{1}{Q_{e}}=\frac{1}{Q_{m}K_{L}}•\frac{1}{C_{e}}+\frac{1}{Q_{m}}$$

where 1/*Q_e_* is the reciprocal of the unit adsorption amount of the adsorbent at equilibrium (g/mg); 1/*C_e_* is the reciprocal of the metal ion concentration in the solution when the adsorption equilibrium is reached (L/mg); *Q_m_* is the single-layer adsorption capacity per unit of adsorbent material (mg/g); and *K_L_* is the Langmuir adsorption constant (L/mg).

The Freundlich model assumes that the adsorption of molecules or metal ions on the surface of the adsorbent is heterogeneous and multilayered. The Freundlich model is described as follows based on an equivalent transformation [39]:

(2) $$\ln(Q_{e})=\frac{1}{n}\ln(C_{e})+\ln k_{f}$$

where ln(*Q_e_*) is the ordinate; ln(*C_e_*) is the abscissa; *k_f_* is a constant associated with the adsorption capacity; and 1/*n* is an empirical constant associated with the adsorption strength that can be determined from the phase regression equation.

#### 2.2.3. Soil Experiment

For the soil experiment, 100 g of soil was weighed into a 750 mL polypropylene plastic box. Montmorillonite, sepiolite, and attapulgite with mass ratios of 2%, 5%, 10%, and 15% were added as immobilization materials. A blank control group (containing no immobilization reagent) was concurrently maintained at a 50% moisture content for two weeks. Subsequently, the soil was air-dried and pulverized through a 2 mm screen under natural ventilation conditions, followed by toxicity leaching experiments using the acetic acid buffer solution method. The extraction liquid was 5.7 mL 99–100% acetic acid and 64.3 mL 1 M NaOH in 1 L Milli-Q water (pH 4.93 ± 0.05), the liquid-solid ratio (volume-to-mass ratio) was set to 20:1, and the solution was shaken at 30 r/min for 18 h using a flip oscillator and left standing for 30 min. The supernatant was filtered using a vacuum filter with 0.8 μm membrane, and the extract was then collected to measure the concentration of the pollutant (China, HJ/T 300-2007) [40]. The effects of the immobilized materialson the physical and chemical properties of the soil were examined. All experiments were conducted in duplicate.

A pH meter (Mettler Toledo, Zurich, Switzerland) was used to measure the pH of the aqueous solution and soil. The amount of cation exchange in the soil was measured using the Hexamminecobalt trichloride solution—Spectrophotometric Method (China, HJ 889-2017) while the organic matter content in the soil was measured using the Potassium Dichromate Oxidation Spectrophotometric method (China, HJ 615-2011) [41,42]. The soil redox potential (Eh) was determined according to China national standard SL 94-1994, using a SX731 portable redox potential tester (Sanxin, Shanghai, China). The mineral material characteristics were analyzed using XRD with Cu-K_α_ radiation (λ = 0.15418 nm) (6000, Shimadzu Corporation, Kyoto, Japan) and a FTIR spectrometer equipped with a deuterated triglycine sulfate (DTGS) detector (Nexus 5DXC, Thermo Nicolet, Waltham, MA, USA). The sample was prepared using the tableting and KBr pellet method respectively. The scanning parameters of XRD were as follows: voltage/current, 40 kV/40 mA; scanning range, 0–90°; scanning step, 0.02°; scanning speed, 10° min^−1^. The parameters setting of FTIR were as follows: resolution, 4 cm^−1^; number of scans, 30 times. The specific surface areas and internal microporous properties of the three mineral materials were analyzed using the BET method via N_2_ adsorption–desorption at 77 K on an ASAP 2460 surface area analyzer (Micromeritics, Norcross, Georgia, USA). A degassing pretreatment (degas temperature, 423 K) was carried out for the samples. The specific surface areas can, then, be calculated from the BET equation. The parameters setting for the BET method were as follows: analysis of adsorption gas, N_2_; analytical bath temperature, 77 K; equilibrium interval, 30 s; sample density, 1.00 g/cm^3^. The SPSS (18.0, SPSS lnc., Chicago, IL, USA) and Origin (8.1, OriginLab, North Ampton, NC, USA) statistical analysis software packages were used for data analysis and the production of charts, respectively.

## 3. Results and Discussion

### 3.1. XRD Analysis

**Figure 1:**
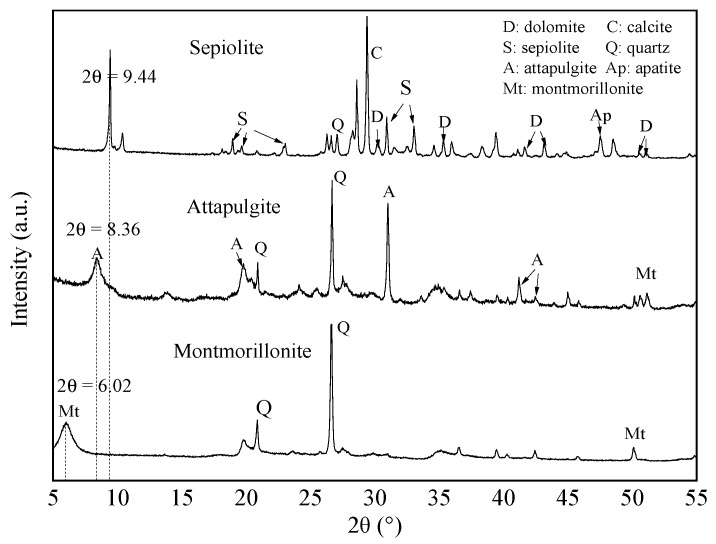
X-ray diffraction pattern analyses of sepiolite, attapulgite, and montmorillonite.

The composition and phase of the mineral materials were analyzed and judged using an XRD analyzer, and the results are shown in Figure 1. Among the three mineral materials, the mineral composition of sepiolite was the most complex, comprising sepiolite, dolomite, calcite, apatite, and quartz. Attapulgite was mainly composed of attapulgite, quartz, and montmorillonite. The mineral components of montmorillonite mainly included quartz and montmorillonite, which had relatively few mineral impurities and the highest purity of the three mineral materials.

The basic layer spacing, D_001_, of the mineral materials was used to measure the eigenvalues of the basic structural units of the mineral materials. Larger layer spacing values correspond to an easier exchange of interlayer ions with external ions [43]. D_001_ can be calculated using the Bragg equation:

(3) $$D_{001}=\frac{1.5418}{2\sin\left(\frac{2\theta }{2}\right)}$$

where D_001_ is the base layer spacing of the mineral material and θ is the incident angle of the X-ray.

The results show that the basic layer spacing values of sepiolite, attapulgite, and montmorillonite were 9.37, 10.57, and 14.68 Å, respectively. The montmorillonite was Ca^2+^-montmorillonite and had the largest unit interlayer spacing of the three mineral materials, indicating that it has the potential to accommodate more metal ions or other molecules.

### 3.2. FTIR Spectral Analysis

**Figure 2:**
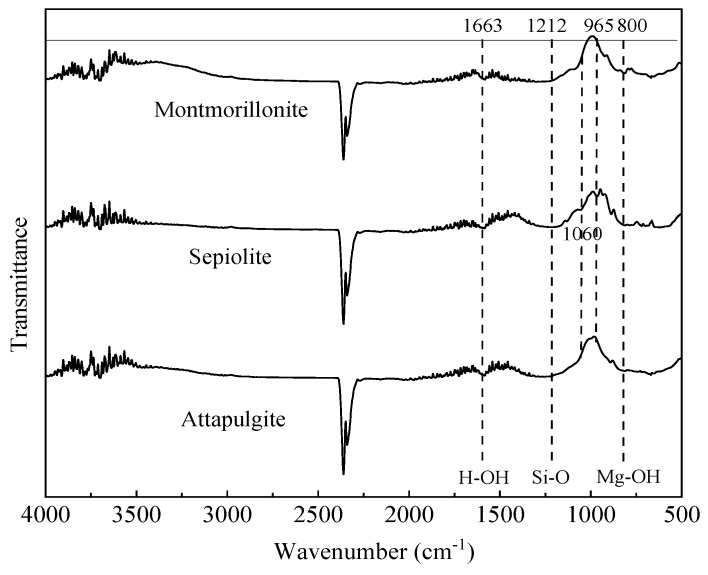
Fourier transform infrared spectral pattern analyses of sepiolite, attapulgite, and montmorillonite.

The FTIR results (Figure 2) show that the surface molecular bonds of the three mineral materials were similar. The peak at a wavenumber of 1663 cm^−1^ reflects the presence of adsorbed and zeolitic water in the mineral material layers. The changes in transmittance at 1212 and 965 cm^−1^ are related to the stretching and bending vibration of the Si–O bond, whereas transmittance changes at 800 cm^−1^ can be attributed to the stretching vibration of Mg-OH. The changes in transmittance observed at 1060 cm^−1^ is caused by PO_4_^3−^ vibration, which indicates the possible presence of apatite in the sepiolite [44,45]. Compared with montmorillonite and attapulgite, more molecular groups occurred on the surface of sepiolite. In general, the FTIR spectra indicate that Mg–OH and Si–O bonds were mainly present in the interlayer structure of the three minerals.

### 3.3. BET Analysis

Table 2 lists the specific surface parameters. The results show that the order of the specific surface areas of the three mineral materials was as follows: attapulgite > montmorillonite > sepiolite. The specific surface area of sepiolite was significantly smaller than that of attapulgite or montmorillonite. The unit pore volumes showed the same trend. Larger specific surface areas and unit pore volumes tend to correspond to more adsorption sites and greater load capacities. Therefore, materials with these properties generally show better removal effects on heavy metal ions.

### 3.4. Removal of Cr(VI) from Aqueous Solution Using Mineral Materials

#### 3.4.1. Removal of Cr(VI) from Aqueous Solution by Mineral Materials

**Figure 3:**
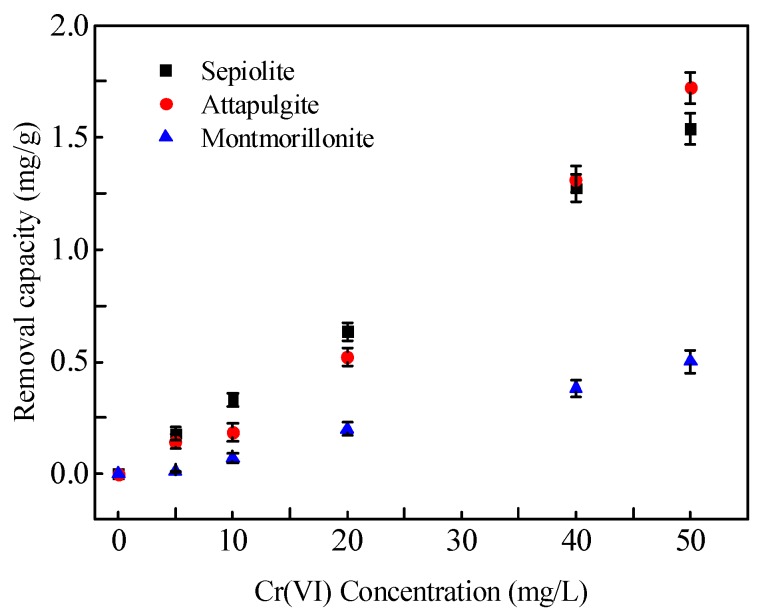
Removal of Cr(VI) from solution by three mineral materials (solid/liquid = 1:100; solution pH = 7.2).

The results of the removal of Cr in aqueous solution by the three mineral materials are shown in Figure 3. The results indicated that the removal ability of all three mineral materials of Cr(VI) in the solution increased with the increase in the Cr(VI) concentration. Sepiolite and attapulgite had a better removal effect of Cr(VI) in solution than montmorillonite, which may result from these two mineral materials containing more mineral impurities such as calcite, sepiolite, and dolomite. The carbonate component of these mineral impurities has a strong adsorption and complexation effect on Cr(VI). In addition, the pH of the three mineral materials was in the following order: sepiolite > attapulgite > montmorillonite, which was consistent with the removal potential of Cr(VI) by the three minerals at the concentration of 0–20 mg/L. Adsorption was the main reason for the removal from Cr(VI) in the solution when the Cr(VI) concentration was below 20 mg/L. As the Cr(VI) concentration increased, the ability of attapulgite to remove Cr(VI) from solution was better than that of sepiolite, which may be attributed to the fact that the layer spacing of the former was larger than that of the latter. Ion exchange contributed greatly to the removal of Cr(VI) from the solution.

#### 3.4.2. Adsorption Isotherms

**Figure 4:**
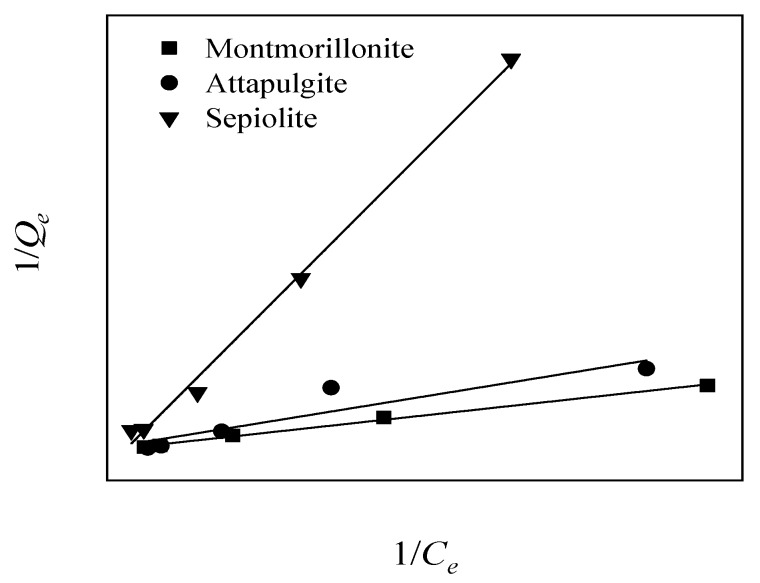
Langmuir isotherm adsorption model of the three mineral materials for Cr(VI) ion adsorption in solution (solid/liquid = 1:100; mineral material addition amount = 500 mg; pH = 7.2; temperature = 25 °C).

**Figure 5:**
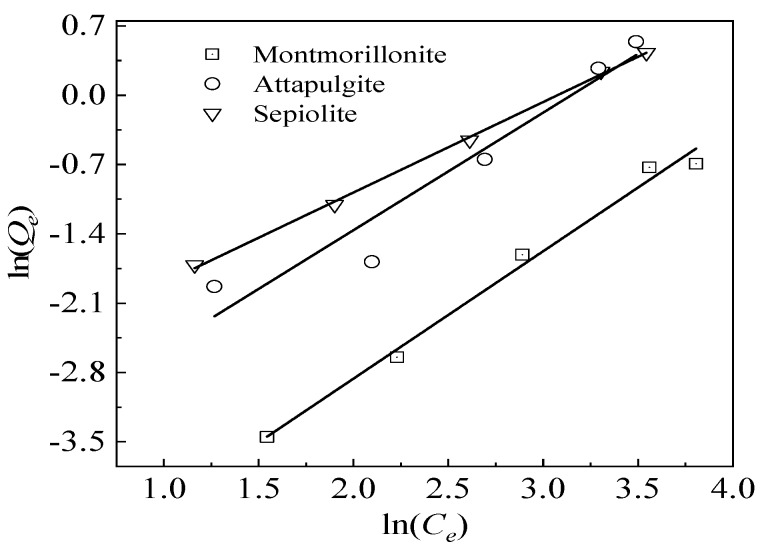
Freundlich isotherm adsorption model of the three mineral materials for Cr(VI) ion adsorption in solution (solid/liquid = 1:100; mineral material addition amount = 500 mg; pH = 7.2; temperature = 25 °C).

The adsorption data for the Cr(VI) ions in the three mineral materials at 25 °C were substituted into the Langmuir and Freundlich isotherm adsorption model. Figure 4 and Figure 5 show these results, whereas Table 3 lists the values of the relevant parameters in the two isotherm adsorption models. 

By comparing and analyzing the results of the isothermal adsorption model fitting of the aqueous Cr(VI) concentrations after adsorption by the three mineral materials, we found that sepiolite had a good fitting effect for these two isothermal adsorption models (R^2^ > 0.995, *p* < 0.01), which may have been a result of the low Cr(VI) concentrations in the 0.25–2.5 mg solution used for the adsorption experiment. However, the single-layer adsorption process was not thorough. Attapulgite showed a higher degree of fitting to the Freundlich isotherm adsorption model, in that the R^2^ of the Freundlich isotherm (0.9355, *p* < 0.01) was higher than that of the Langmuir isotherm adsorption model (R^2^ = 0.8591, *p* < 0.05), indicating that the attapulgite adsorption process for Cr(VI) was multilayered. In addition, the fibrous morphologies of attapulgite/sepiolite contributed to the adsorption of Cr as well, and which can further load more modifiers to improve its adsorption performance. Montmorillonite had a higher degree of fitting to the Langmuir model (R^2^ = 0.9955, *p* < 0.01), indicating that the adsorption of Cr(VI) on the montmorillonite surface occurred at a specific single-phase point. 

#### 3.4.3. Effect of pH on Adsorption

**Figure 6:**
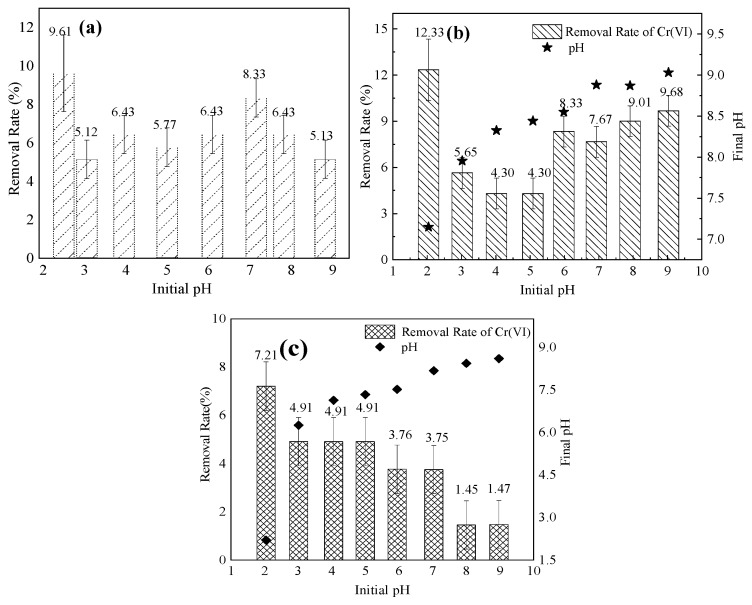
Effect of pH on the three mineral materials: (**a**) attapulgite, (**b**) sepiolite, and (**c**) montmorillonite for Cr(VI) removal from an aqueous solution (mineral material concentration = 10 g/L; solution volume = 50 mL; initial Cr(VI) concentration = 30 mg/L).

The pH significantly affects the adsorption of heavy metals; H^+^ or OH^−^ in an aqueous solution changes the zeta potential on the surface of the mineral material, thereby affecting the electrostatic adsorption process. Furthermore, H^+^ or OH^−^ in a solution occupies the active potential of the bio-adsorbed surfaces of the mineral materials, which competes with the adsorption of metal ions [46,47]. In this study, we examined the effects of the three mineral materials on the adsorption of Cr(VI) in solution at pHs ranging from 2–9 (Figure 6). Attapulgite (Figure 6a) showed the highest removal rate (approximately 9.6%) for aqueous Cr(VI) at a pH of 2.5. When the pH increased to 7, the effect of attapulgite on the removal of aqueous Cr(VI) was not significantly affected, whereas there was a reduction in the removal rate of Cr(VI) with attapulgite at pHs between 8 and 9. The removal rate of aqueous Cr(VI) by sepiolite (Figure 6b) was 16.39% at a pH of 2. When the pH increased from 2 to 5, the removal rate gradually decreased. When the pH increased from 5 to 9, there was a slight increase in the Cr(VI) removal rate. Figure 6c shows the effect of pH on aqueous Cr(VI) removal by montmorillonite. The removal rate of aqueous Cr(VI) was 7.9% at a pH of 2. Then, when the pH increased, there was a decrease in the removal rate of aqueous Cr(VI) by montmorillonite.

Cr(VI) mainly exists in the form of CrO_4_^2−^ in acid environments and HCrO_4_^−^ in alkaline environments [48]. The adsorption of CrO_4_^2−^ and HCrO_4_^−^ by three minerals was related to the surface charge of clay minerals in different pH environments. The effect of pH on aqueous Cr(VI) removal by attapulgite occurred as OH^−^ stacking near the surface of the mineral material as the pH increased. Therefore, the surface of the material became negatively charged, while reducing the electrostatic adsorption of negatively charged aqueous ionic groups, such as CrO_4_^2−^ and HCrO_4_^−^ [49]. In addition, under alkaline conditions, two adsorption sites for CrO_4_^2−^ contributed significantly to the decrease in the aqueous Cr(VI) removal rate [50]. Sepiolite has a small specific surface area, and, under acidic conditions, some OH^−^ on the surface of sepiolite was neutralized. The contact rate between CrO_4_^2−^, HCrO_4_^−^, and the materials simultaneously decreased with an increase in pH from 2 to 5, such that the Cr(VI) removal rate was higher at a pH of 2. Surface group complexation was the main reason for the increased aqueous Cr(VI) removal rate, as the pH increased. Additionally, the CaCO_3_/MgCO_3_ in sepiolite released cations under low pH conditions, which also contributed to the adsorption of CrO_4_^2−^ by materials. The removal of aqueous Cr(VI) from the solution by montmorillonite mainly depended on adsorption, whereas the initial pH environment of the solution affected the surface charge characteristics of the mineral. Therefore, when the surface of the material changed from positively to negatively charged, there was a reduction in the CrO_4_^2−^ and HCrO_4_^−^ removal ability of montmorillonite.

### 3.5. Cr-Contaminated Soil Remediation by the Mineral Materials

#### 3.5.1. Cr(VI) Immobilization Effect

**Figure 7:**
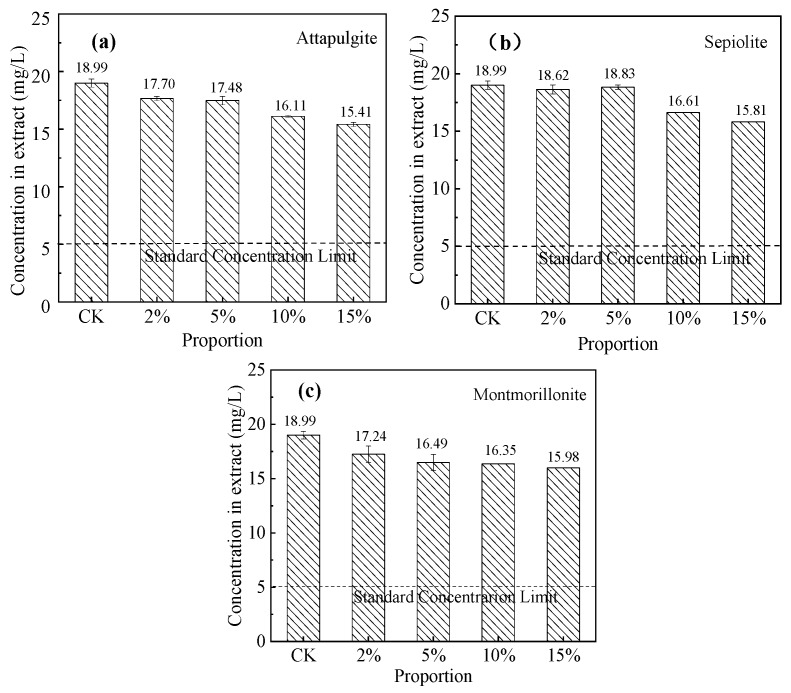
Effect of mineral materials on Cr(VI) immobilization in soil: (**a**) attapulgite, (**b**) sepiolite, and (**c**) montmorillonite. CK: control check. (initial Cr(VI) concentration in soil = 718.05 mg/kg).

We evaluated the Cr(VI) immobilization effects of sepiolite, attapulgite, and montmorillonite in soil (Figure 7). The results show that increasing the amounts of mineral materials decreased the leaching concentrations of Cr(VI) in the soil. However, when the proportion of sepiolite was < 5%, there was no reduction in the Cr(VI) concentration of the leachate. Immediately following the addition of attapulgite or montmorillonite to the soil, the Cr(VI) in the soil was rapidly and effectively immobilized. With the addition of 15% sepiolite, attapulgite, or montmorillonite, the Cr(VI) concentrations in the leachates decreased by 16.8%, 18.9%, and 15.9%, respectively. Of the three mineral materials, attapulgite had the clearest Cr(VI) immobilization effect in the contaminated soil, which can be attributed to the lower pH of attapulgite (6.74) compared with that of sepiolite (8.59). Hence, we observe that attapulgite had a strong adsorption capacity. In addition, the XRD results confirmed that attapulgite contained more complex mineral impurity components (such as dolomite and calcite) as compared with montmorillonite, and its surface can thus provide more molecular groups that can adsorb or react with CrO_4_^2−^ and HCrO_4_^−^. To a certain extent, the three mineral materials can remediate Cr(VI)-contaminated soil. However, even when the concentration of a single mineral material in soil reached 15%, the contaminated soil was still considered hazardous waste, because the Cr(VI) concentration limit of the toxicity characteristic leaching procedure is 5 mg/L. In conclusion, the addition of a single natural mineral material can only reduce Cr(VI) pollution to a certain extent, which does not conform to environmental remediation targets.

#### 3.5.2. Total Cr Immobilization Effect

**Figure 8:**
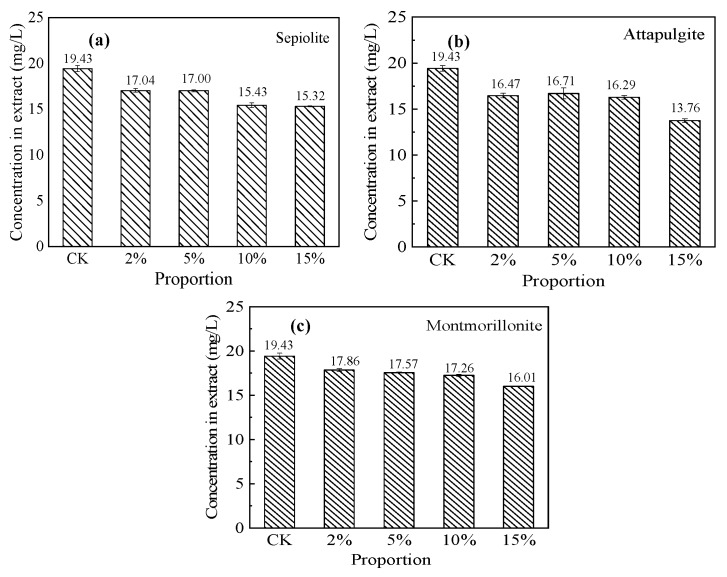
Immobilization effects of the three mineral materials on total Cr in (**a**) sepiolite-, (**b**) attapulgite-, and (**c**) montmorillonite-treated soils. CK: control check. (initial total Cr concentration in soil = 718.05 mg/kg).

As shown in Figure 8, the total Cr concentration in the blank soil leachate was 19.43 ± 0.3 mg/L. Following a 2% addition of each of the three mineral materials, there was a slight reduction of the total Cr concentration in the soil extract. The decreasing amplitudes of the total soil extract Cr concentration of the sepiolite and attapulgite groups were greater than that of the montmorillonite group. When the proportion of mineral materials was increased to 10%, there was a large reduction in the total Cr concentration of the contaminated soil extracts. With the addition of 15% sepiolite, attapulgite, and montmorillonite, the total Cr concentrations were reduced by 21.2%, 29.2%, and 17.6%, respectively. The Cr(VI) in the immobilized soil was mainly adsorbed at a specific point, whereas the immobilization of Cr(III) mainly occurred via electrostatic attraction and specific adsorption mechanisms [33]. This indicates that attapulgite had a stronger adsorption capacity for exchangeable Cr(III) and Cr(VI) in soil.

### 3.6. Effect of Mineral Materials on Soil pH

Table 4 lists the effects of the mineral materials on the soil pH. The pH of the original soil was 8.16, whereas that of sepiolite, attapulgite, and montmorillonite was 8.59, 6.74, and 8.02, respectively. After adding certain proportions of sepiolite, attapulgite, and montmorillonite to the soil, the soil pH slightly reduced after remediation. This was mainly because the CrO_4_^2−^ in the soil produced OH^−^ during hydrolysis and contributed to the alkalinity, whereas the addition of mineral materials led to the combination of CrO_4_^2−^ and OH^−^ groups on the surface of the mineral materials, reducing hydrolysis. Attapulgite had the most significant influence on soil pH because its low pH neutralized a portion of the alkalinity. Moreover, attapulgite had the overall strongest immobilization effect on Cr(VI), adsorbing CrO_4_^2−^ in the soil and lowering the soil pH. Therefore, we further confirmed, based on the change in the soil pH, that attapulgite had the strongest effect on Cr immobilization in the soil of the three mineral materials.

## 4. Conclusions

The purities of the three mineral materials decreased as montmorillonite > attapulgite > sepiolite, as well as having similar surface molecular bond types. The order of the aqueous Cr(VI) ion adsorption potential of the three mineral materials was sepiolite > attapulgite > montmorillonite. When the concentration of aqueous Cr(VI) was 0–50 mg/L, the adsorption of Cr(VI) by montmorillonite occurred in a single-layer specific-point manner, whereas the adsorption of Cr(VI) by attapulgite was a multilayer adsorption process. Both single and multilayer adsorption models described the adsorption behavior of Cr(VI) to sepiolite (R^2^ > 0.99). The pH had an effect on Cr(VI) removal by the mineral materials. For montmorillonite and attapulgite, a lower pH corresponded to higher Cr(VI) removal effects. For sepiolite, the aqueous Cr(VI) removal effect was lowest when the pH was approximately 5.0. In the soil experiments, following the addition of 15% sepiolite, attapulgite, and montmorillonite, the Cr(VI) concentrations in the leachates decreased by 16.8%, 18.9%, and 15.9%, respectively, whereas the total Cr concentrations in the leachates decreased by 21.2%, 29.2%, and 17.6%, respectively. Among the three mineral materials, attapulgite had the strongest effect on Cr immobilization in soil, as well as beneficial effects on the soil pH. Therefore, attapulgite can be employed as an effective environmental material for the immobilization and removal of Cr(VI) as an effective environmental material. The results of this study revealed the adsorption mechanisms and provided insights regarding the immobilization of Cr(VI) in water and soil by natural mineral materials.

## Figures and Tables

**Table 1:** Physical and chemical properties of the soil used in this study.

Soil Properties	Redox Potential (mV)	Organic Matter (g/kg)	Cation Exchange Capacity (cmol/kg)	pH	Cr(VI) Content (mg/kg)	Total Cr Content (mg/kg)
Test values	227 ± 21	15.6 ± 3	26.0 ± 4	8.16 ± 0.02	424.59 ±17	718.05 ± 28

**Table 2:** Brunauer–Emmett–Teller (BET) analysis of the three mineral materials.

Mineral Material	BET Specific Surface Area (m^2^/g)	Pore Volume (cm^3^/g)
Sepiolite	5.73 ± 0.05	0.018 ± 0.03
Attapulgite	119.82 ± 0.1	0.31 ± 0.05
Montmorillonite	84.24 ± 0.08	0.11 ± 0.02

**Table 3:** The Freundlich and Langmuir isotherm adsorption model parameters of the Cr6+ on the three mineral materials.

Mineral Material	Langmuir Model			Freundlich Model		
	*K_L_* (L/mg)	*Q_m_* (mg/g)	R^2^	*K_F_* (mg^1−1/*n*^L^1/*n*^/g)	*n*	R^2^
Sepiolite	0.0133	4.35	0.9961 ^**^	21.54	0.92	0.9988 ^**^
Attapulgite	0.0133	2.94	0.8594 ^*^	22.42	1.27	0.9355 ^**^
Montmorillonite	0.0162	0.39	0.9955 ^**^	67.36	1.30	0.9904 ^**^

*K_L_*: Langmuir adsorption constant; *Q_m_*: single-layer adsorption capacity per unit of adsorbent material; *K_F_*: constant associated with the adsorption capacity; *n*: constant associated with the adsorption strength; R^2^: correlation coefficient; *: significant correlation found at 0.05 level; **: significant correlation found at 0.01 level.

**Table 4:** Effects of the addition of mineral materials on soil pH.

Material	pH	Group	pH
Untreated Soil	8.16	Blank	8.16
Sepiolite	8.59	Soil + 5% Sepiolite	8.10 ± 0.02
		Soil + 10% Sepiolite	8.09 ± 0.02
		Soil + 15% Sepiolite	8.09 ± 0.02
Attapulgite	6.74	Soil + 5% Attapulgite	8.09 ± 0.02
		Soil + 10% Attapulgite	7.97 ± 0.02
		Soil + 15% Attapulgite	7.96 ± 0.02
Montmorillonite	8.02	Soil + 5% Montmorillonite	8.01 ± 0.02
		Soil + 10% Montmorillonite	8.03 ± 0.03
		Soil + 15% Montmorillonite	8.06 ± 0.02

## Abbreviations

XRD	X-ray diffraction
FTIR	Fourier-transform infrared
BET	Brunauer–Emmett–Teller
